# Genome-wide identification and characterization of the Groucho/Tup1-like corepressor family identifies a potential role in the epigenetic regulation of abiotic stress responses in soybean

**DOI:** 10.3389/fpls.2026.1825108

**Published:** 2026-06-04

**Authors:** Ziqi Zhang, Xintong Han, Xingfu Yin, Yang Zhang, Yinji Jiang, Yongyi Li, Weilin Wu, Songquan Wu, Zhuo Jin, Mingzhe Shen

**Affiliations:** 1 College of Agriculture, Yanbian University, Yanji, China; 2 Yanbian Academy of Agricultural Sciences, Longjing, China

**Keywords:** abiotic stresses, expression pattern, genome-wide identification, groucho/tup1-like corepressor, histone H3 acetylation, protein interaction, soybean

## Abstract

The Groucho/Tup1 family of corepressors is known to play a role in fine-tuning gene expression in eukaryotes by recruiting key epigenetic regulators, histone deacetylases (HDACs), to chromatin. In plants, members of this family regulate growth, development, and responses to stress. However, their homologs in soybean (*Glycine max*(L.) Merr.) have not been systematically characterized, and their roles in adapting to abiotic stress remain largely uncharacterized. In this study, we identified 39 putative Groucho/Tup1-like corepressor (*GmGTLC*) genes in the soybean genome, distributed across 16 chromosomes. Phylogenetic analysis classified the GmGTLC proteins into six subfamilies, with members within each subfamily exhibiting conserved gene and protein structures. Promoter analysis revealed multiple cis-regulatory elements associated with light responsiveness, hormone signaling, and responses to abiotic stress. Gene duplication analysis indicated that segmental duplication events were the primary driver of *GmGTLC* family expansion. Expression profiling showed that *GmGTLC* genes are broadly expressed in roots, hypocotyls, cotyledons, tender leaves, mature leaves, unopened flowers, open flowers, pods, and seeds. Furthermore, RT-qPCR analysis of soybean seedlings subjected to cold (4 C for 0, 3, 6, 12, and 24h), salt (200 mM NaCl for 0, 3, 6, 12, and 24h), or drought (air-drying for 0, 0.5, 1, 3, and 6h) stress indicated that most tested genes were induced by cold. While gene-specific expression patterns were observed under salt and drought conditions. Western blot assays showed that global histone H3 acetylation levels were elevated under abiotic stress conditions, providing correlative evidence of a link between this epigenetic modification and stress adaptation. Protein interaction assays in a heterologous transient system (*N. benthamiana*) suggested a potential interaction between GmHOS15A/D and two GmHDAC family proteins, GmHDA12 and GmHDA13, indicating an association that may be relevant to histone deacetylation. Collectively, this study provides a comprehensive characterization of the *GmGTLC* gene family at the genomic and expression levels. The exploratory and correlative findings suggest a potential role for GmHOS15A/D in epigenetic regulation of abiotic stress responses in soybean, laying a preliminary foundation for future mechanistic studies and molecular breeding strategies.

## Introduction

Transcriptional regulation is a complex and pivotal biological process that integrates and interprets diverse intracellular and extracellular signals to determine both the temporal and spatial cell identity and functionality during plant growth, development, and environmental responses (Casamassimi and Ciccodicola, 2019; Zhang et al., 2022). Although this process largely depends on transcription factors (TFs), it is not solely controlled by them. Indeed, the coordinated action of multiprotein coregulator complexes, including transcriptional coactivators and corepressors, is also required to fine-tune gene expression dynamics. Coactivators facilitate transcription by recruiting multiprotein complexes that modify epigenetic marks, such as histone acetylation and demethylation, thereby promoting efficient gene expression. In contrast, corepressors associate with the transcriptional machinery and typically mediate histone deacetylation and methylation, leading to transcriptional repression (Talukdar and Chatterji, 2023).

Transcriptional corepressors are ubiquitous in eukaryotic cells that function as molecular scaffolds that recruit chromatin-modifying enzymes such as histone deacetylases (HDACs), thereby regulating histone deacetylation, modulating chromatin compaction, and ultimately repressing transcription (Perissi et al., 2010). For instance, the well-characterized Groucho/Tup1 corepressor family belongs to this category, including Drosophila Groucho (Gro), yeast thymidine uptake 1 (Tup1), mammalian transducing-like enhancer of split (TLE), and Arabidopsis TOPLESS(TPL), LEUNIG(LUG), and high expression of osmotically responsive gene 15 (HOS15) (Wickner, 1974; Sasai et al., 1992; Conner and Liu, 2000; Flores-Saaib and Courey, 2000; Long et al., 2002; Zhu et al., 2008).

The Groucho/Tup1 corepressor family is a group of evolutionarily conserved transcriptional regulators (Chen and Courey, 2000; Copley, 2005; Liu and Karmarkar, 2008). The members share a similar domain architecture and functional properties. Gro, initially characterized in *Drosophila*, and its vertebrate ortholog TLE, contains a distinctive N-terminal glutamine (Q)-rich domain and a highly conserved C-terminal WD40 repeat domain. The Q-rich domain primarily mediates tetramerization and perhaps higher-order oligomers and facilitates the binding of a subset to recruiting TFs. The WD40 domain facilitates interactions with a broader range of TFs and other proteins. Although yeast Tup1 does not show strong sequence homology to Gro, it is considered a functional homolog and classified into the Groucho/Tup1 superfamily based on its WD-repeat domain similarity and corepressor activity (Courey and Jia, 2001). In Arabidopsis, Groucho/Tup1-like corepressor (GTLC) proteins comprise 13 genes. These can be grouped into two separate subfamilies, TPL/TPR (TOPLESS-RELATED) and LUG/LUH (LEUNIG HOMOLOG). Interestingly, in addition to the central Q-rich domain and the C-terminal WD40 repeat domain, Arabidopsis GTLC proteins also contain an N-terminal LisH (lissencephaly homology) domain, which is usually followed by CTLH (C-terminal to LisH) domain, LUFS domain, or F-box-like motifs (Liu and Karmarkar, 2008; Shen et al., 2020). Thus, they form distinct protein-protein interactions to mediate functional specialization.

GTLC proteins play crucial roles in the diverse biological processes, spanning developmental regulation and environmental stress responses in plants. LUG represents the first characterized member of this family in Arabidopsis (Liu and Meyerowitz, 1995). The LUG subfamily consists of two functionally related proteins, LUG and LUH, which exhibit partially redundant functions across multiple developmental processes (Liu and Karmarkar, 2008; Sitaraman et al., 2008). These include floral and embryonic development, microsporocyte formation, ovule outer integument development, seed coat mucilage extrusion, leaf polarity maintenance, and meristem activity regulation (Lee et al., 2015; Shi et al., 2024; Simon et al., 2017; Sitaraman et al., 2008; Stahle et al., 2009). TPL/TPRs participate in a broad range of biological processes, including hormone signaling, embryogenesis, organ growth, reproduction, circadian regulation, and responses to biotic and abiotic stresses (Plant et al., 2021; Saini and Nandi, 2022). HOS15 also functions as a GTLC with unique features, participating in the regulation of plant developmental processes and proteins involved in biotic/abiotic stress responses (Ali et al., 2025). In rice (*Oryza sativa*), the TPL homologs ASP1 and OsREL2 regulate sterile lemma identity (Wu et al., 2021; Yoshida et al., 2012). OsTPL/TPR negatively regulates long-distance iron (Fe) transport, facilitating adaptation to fluctuating Fe availability and maintaining Fe homeostasis (Wang et al., 2022). Furthermore, OsTPL/TPR synergistically enhances resistance to the brown planthopper (BPH) by transcriptionally repressing *F3’H* (Sun et al., 2023). Separately, the OsLUGL (LEUNIG-like) participates in a complex with OsSEU, OsAP1, and OsSEP3 that targets the *OsGH3–8* promoter. This modulates auxin levels and *OsARFs* expression. Thereby influencing floral development (Yang et al., 2019). In maize (*Zea mays*), the TPL-related REL2 family governs development from embryogenesis through reproductive growth and shows potential for enhancing seed yield (Gregory et al., 2024). Additionally, in wheat (*Triticum aestivum* L.), TaTPR1 positively regulates post-penetration resistance against powdery mildew, likely through suppressing the susceptibility (S) genes *TaDND1* and *TaDND2* (Zhi et al., 2024). TaHOS15 interacts with TaHDA6, forming a histone deacetylase complex to fine-tune the defense response to *Blumeria gramini* f.sp*. tritici* (Bgt) (Liu et al., 2019).

Soybean (*Glycine max* (L.) Merr.) is a globally significant agricultural crop, valued as a key source for humans and animals due to its high oil and protein content. However, soybean production faces substantial yield losses from environmental stresses. Drought stress can cause losses of more than 50% (Arya et al., 2021), cold stress typically reduces seed yield by approximately 24% (Staniak et al., 2021), and salinity stress, to which soybean exhibits moderate sensitivity, leads to yield reductions of 20–40% (Guan et al., 2014; Papiernik et al., 2005). Histone modification, a form of epigenetic regulation, plays an essential role in regulating gene expression and enhancing environmental adaptability during soybean adaptation to abiotic stresses. Under cold stress, the levels of H3K4me3, H3K9ac, and H4K12ac decrease in root tip cells, whereas the level of H3K9me2 increases. These changes are reversible upon recovery, suggesting the existence of a flexible chromatin-based mechanism for cold adaptation (Stępiński, 2012). Under salt stress, pretreatment induces genome-wide reprogramming of H3K4me2/3 and H3K9ac, a process that suppresses defense pathways while coordinating ion homeostasis and osmotic regulation (Yung et al., 2022). The salt-induced upregulation of four transcription factor genes is likely mediated by increased H3K4me3 and decreased H3K9me2, suggesting that these chromatin modifications contribute to enhanced salinity tolerance in plants (Song et al., 2012). Under drought stress, overexpressing *GmUBC9* enhances H2B monoubiquitination (H2Bub1), thereby activating downstream drought-responsive genes and improving tolerance (Chen et al., 2020). Thus, histone modifications represent promising epigenetic targets for breeding climate-resilient soybeans. It is therefore essential to identify key genes that balance growth and stress adaptation by regulating histone modifications, thereby unlocking yield potential under environmental stresses.

In plants, the GTLC proteins were primarily characterized in Arabidopsis. Although studies on other crop species have emerged in recent years (Guan et al., 2018; Hao et al., 2014; Li et al., 2016; Zhang et al., 2024), a comprehensive exploration of the *GTLC* gene family in soybean remains lacking. Therefore, in this work, we conducted a comprehensive genome-wide investigation of *GTLC* genes in soybean (*GmGTLCs*), analyzed their stress-responsive expression profiles, and explored the interactions between representative members of the GmHOS15s and two GmHDAC proteins to elucidate their potential role as transcriptional corepressors. Our findings establish a foundation for elucidating the potential biological functions of the GmGTLC family, which may be involved in regulating histone deacetylation, and provide a theoretical basis for improving future soybean yield and stress tolerance through molecular breeding.

## Materials and methods

### Identification of candidate *GmGTLC* genes in soybean

The soybean genome assembly (*Glycine max* Wm82.a6.v1, Phytozome genome ID: 880) was retrieved from the JGI database (https://phytozome-next.jgi.doe.gov/info/Gmax_Wm82_a6_v1, accessed on 10 August 2025) (Goodstein et al., 2012). To identify candidate *GmGTLC* genes, Hidden Markov model (HMM) profiles for the LisH (PF08513) and WD40 repeat (PF00400) domains were obtained from the Pfam database (http://pfam-legacy.xfam.org/, accessed on 16 August 2025) (Mistry et al., 2021). Candidate GmGTLC proteins were initially identified by performing hmmsearch (Simple HMM Search tools from TBTools-II) against the soybean proteome with an E-value cutoff of 1e^-5^, complemented by BLASTp searches with the 13 *Arabidopsis thaliana* GTLC proteins (an E-value cutoff of 1e^-5^). All retrieved sequences were further validated for the presence of both conserved domains using NCBI CD-Search (https://www.ncbi.nlm.nih.gov/Structure/cdd/wrpsb.cgi, accessed on 5 September 2025) and SMART (https://smart.embl.de, accessed on 5 September 2025). Redundant or truncated sequences lacking essential domain architecture were manually removed. The final non-redundant set of high-confidence *GmGTLC* genes was used for downstream analyses.

### Characterization of chromosomal location, conserved domain, and structure distribution of GmGTLCs

The chromosomal positions of *GmGTLC* genes were extracted from the *Glycine max* Wm82.a6.v1 GFF3 annotation file and visualized using the “Gene Location Visualize from GTF/GFF” tool in TBtools-II (v2.390). Gene structures, reflecting exon–intron organization, were determined based on the GFF3 file. Conserved protein domains were validated via NCBI CD-Search, while additional motifs were identified using MEME Suite (https://meme-suite.org/meme/, accessed on 10 September 2025). A phylogenetic tree was constructed from a Clustal Omega alignment (EMBL-EBI) using IQ-Tree (via TBtools IQ-Tree Wrapper tool) with 1,000 ultrafast bootstrap replicates. All features—phylogeny, gene structure, motifs, and domains—were integrated into a single schematic using TBtools “Gene Structure View (Advanced)” for comprehensive visualization.

### Phylogenetic classification, physicochemical properties, and subcellular localization of GmGTLCs

To establish phylogenetic relationships, the protein sequences of the Arabidopsis GTLC family were retrieved from TAIR (https://www.arabidopsis.org/, accessed on 8 September 2025). These, together with the soybean GmGTLC sequences, were subjected to multiple sequence alignment using Clustal Omega via the EMBL-EBI web server (https://www.ebi.ac.uk/jdispatcher/msa/clustalo, accessed on 8 September 2025) (Madeira et al., 2024). A maximum-likelihood phylogenetic tree was inferred using IQ-TREE (via the “IQ-Tree Wrapper” in TBtools), with the best-fit substitution model selected by ModelFinder and 1,000 ultrafast bootstrap replicates. The resulting tree was visualized and annotated using iTOL (https://itol.embl.de, accessed on 8 September 2025). Based on their clustering with characterized Arabidopsis GTL clades, the soybean GTLC genes were classified into corresponding subfamilies and systematically renamed accordingly.

The physicochemical properties of GmGTLC proteins were calculated using the “Protein Parameter Calc (ProtParam-based)” tool in TBtools, to determine molecular weight (MW, in Da), isoelectric point (pI), instability index (proteins with index < 40 classified as stable, ≥ 40 as unstable), aliphatic index (indicative of thermostability), and grand average of hydropathy (GRAVY; positive values denote hydrophobicity, negative values hydrophilicity). The number of exons for each GmGTLC was extracted from the Wm82.a6.v1 GFF3 annotation file using custom awk scripts. Subcellular localization was predicted using Plant-mPLoc 2.0 (http://www.csbio.sjtu.edu.cn/bioinf/plant-multi/, accessed on 15 September 2025), a plant-specific multi-label predictor that accounts for proteins potentially localized to multiple cellular compartments.

### *GmGTLC* gene synteny analyses and duplication

Synteny analyses of *GmGTLC* genes were performed using TBtools (“One Step MCScanX”). We examined intra-genomic (soybean self) and inter-genomic comparisons with *Arabidopsis thaliana* (Araport11) and *Oryza sativa* (IRGSP-1.0). Collinear gene pairs were identified under default MCScanX settings (E-value ≤ 1e-5) and visualized using “Dual Synteny Plot” and “Advanced Circos” in TBtools.

To evaluate selective pressure, the “Simple Ka/Ks Calculator” in TBtools was used to calculate Ka, Ks, and Ka/Ks ratios for all collinear *GmGTLC* gene pairs. Ka/Ks < 1, = 1, and > 1 were interpreted as evidence of purifying selection, neutral evolution, and positive selection, respectively.

### Promoter cis-regulatory elements analysis of *GmGTLCs*

The 2,000-bp upstream sequences of each GmGTLC gene were retrieved to serve as the promoter region and were analyzed using the PlantCARE database (http://bioinformatics.psb.ugent.be/webtools/plantcare/html/, accessed on 20 September 2025) to predict cis-regulatory elements (CREs).

Based on their functions, the predicted CREs were classified into four categories: hormone-responsive elements, stress-responsive elements, light-responsive elements, and developmental-related elements. To visualize these features, TBtools was employed to generate two complementary formats: (i) a linear schematic showing the position and type of each CRE relative to the promoter region; and (ii) a clustered heatmap using the “Heatmap” module. In the heatmap, gene rows were arranged to reflect the phylogenetic topology of the GmGTLC proteins described in Section 2.3.

### Expression pattern analysis of *GmGTLC* gene family in tissues

To investigate the spatial expression profiles of *GmGTLC* genes, transcriptome data were retrieved from the JGI Plant Gene Atlas available on SoyBase (https://data.soybase.org/Glycine/max/expression/Wm82.gnm6.ann1.expr.Wm82.Sreedasyam_Plott_2023/, accessed on 10 October 2025). The analysis focused on the *Glycine max* cv. Williams 82 (Wm82.a6.v1) genome assembly. We extracted Transcripts Per Million (TPM) values for eight representative tissue types: seed, shoot tip, roots, leaf, stem, nodules, unopened flower, and opened flower. Heatmaps were generated using TBtools to visualize the tissue-specific expression patterns of the *GmGTLC* gene family.

### Plant materials, growth conditions, and stress treatments

Seeds of the soybean cultivar Williams 82 (Wm82) were germinated and grown in a controlled-environment growth chamber maintained at 23 ± 2°C with 60% relative humidity and an average photon flux density of 250 μmol m^-^² s^-^¹ under long-day (LD) conditions (16-h light/8-h dark photoperiod). For tissue-specific expression analysis, samples were collected at different developmental stages: roots, hypocotyls, and cotyledons were harvested from 7-day-old seedlings; tender leaves were harvested from 15-day-old seedlings; mature leaves, flower buds, and open flowers were collected from 60-day-old plants; fruit pods were sampled at 70 days after planting; and seeds were obtained from 85-day-old plants. For cold stress treatments, Wm82 seeds were sown in pots and cultivated at 23 ± 2°C under LD conditions. After 15 days, seedlings were then subjected to 4°C for 0, 6, 12, and 24 hours. Plant leaves were harvested immediately at each time point and flash-frozen in liquid nitrogen. For drought stress treatment, 15-day-old Wm82 plants were carefully uprooted, their roots were washed to remove residual soil, and subjected to dehydration by air-drying for 0, 1, 3, or 6 hours. Leaf samples were collected at each time point and immediately flash-frozen in liquid nitrogen for subsequent analysis. For salt stress induction, 15-day-old Wm82 plants were exposed to a 200 mM NaCl solution for 0, 6, 12, or 24 hours. Leaf samples were collected at each specified time point.

### RNA extraction and RT-qPCR analysis

Total RNA was extracted from frozen samples using the RNAprep Pure Plant Kit (TIANGEN Biotech, Beijing, China) following the manufacturer’s instructions. First-strand cDNA was synthesized using 1μg of total RNA in a 20 μl reaction volume using the Reverse Transcription System (Promega, Madison, WI, USA). The RT-qPCR analysis was performed using the 2×SYBR Green SuperReal PreMix Plus (TIANGEN Biotech, Beijing, China) on a CFX384 Touch Real-Time PCR Detection System (Bio-Rad, Hercules, CA, USA). The amplification protocol consisted of an initial denaturation step at 95°C for 1min, followed by 45 cycles of denaturation at 95°C for 10 s and annealing at 60°C for 20 s, with a final extension step at 65°C for 5 s and 95°C for 5 s. The *Cons4* gene (BU578186) served as an internal reference for normalization, and relative gene expression levels were calculated using the 2^-ΔΔCt^ method. All the experiments included three biological replicates. Gene-specific primers were designed using Primer 5.0 software, and the primer sequences are provided in **Supplementary Table 13**.

### Nuclear protein extraction and western blot analysis

Nuclear protein extractions of soybean leaves were performed as previously described with minor modifications (Shen et al., 2020). Briefly, 200 mg of frozen powdered soybean leaves were homogenized in two volumes of Honda buffer (25 mM Tris-HCl, pH 7.4, 0.4 M sucrose, 10 mM MgCl_2_, 2.5% Ficoll 400, 5% dextran 40, 10 mM β-mercaptoethanol, and a proteinase inhibitor cocktail). The homogenate was filtered through a 70 μm nylon mesh by centrifugation at 200g for 3min at 4 C. Triton X-100 was added to a final concentration of 0.5% and incubated on ice for 15min. The solution was centrifuged at 1,500g for 5min to pellet cell debris and nuclei. Resuspend the pellet in 1 mL of Honda buffer containing 0.1% Triton X-100 and centrifuge at 200g for 1min to remove large cell fragments. The supernatant was centrifuged at 1,800 g for 5min to pellet nuclei, and the pellet was gently washed twice with nuclei resuspension buffer (20 mM Tris-HCl, pH 7.4, 2.5 mM MgCl_2_, 25% glycerol, 0.2% Triton X-100). This pellet was resuspended in 100 μl of total protein extraction buffer (100 mM Tris-Cl, pH 7.5, 150 mM NaCl, 0.5% NP-40, 1 mM EDTA, 3 mM DTT, and a protease inhibitor cocktail) and centrifuged at 14,000 g for 10min at 4 C to collect nuclear proteins. Western blot analysis was performed using anti-acetyl-Histone H3 and Anti-Histone H3 antibodies (Sigma-Aldrich, 06-599, 06-755), and antigen proteins were detected by chemiluminescence using ECL-detecting reagent (Millipore). After chemiluminescent detection, images were captured using a ChemiDoc imaging system. Quantification was performed using Image Lab software (Bio-Rad) on four independent biological replicates. Acetylated H3 levels were normalized to total H3 for each replicate, and the normalized value of the untreated control was set to 1. Fold changes for treated samples (cold, salt, drought) were then calculated relative to this control.

### Luciferase complementation imaging assays

For luciferase complementation imaging (LCI) assays, the full-length coding sequences (CDSs) of the target genes were PCR-amplified from cDNA of the soybean cultivar Wm82 using gene-specific primers. Then the full-length sequences of *GmHOS15A*, *GmHOS15D*, *GmHDA12*, and *GmHDA13* were first cloned into the pDONR zeo vector via Gateway^®^ BP reaction (Invitrogen). Subsequently, these sequences were transferred in-frame into the pCAMBIA1300-GWCLuc or pCAMBIA1300-NLucGW destination vectors using Gateway^®^ LR reaction (Invitrogen). The LCI assay was performed as previously described (Chen et al., 2008) with minor modifications. The indicated plasmid constructs were transformed into *Agrobacterium tumefaciens* strain GV3101. Bacteria were cultured in LB medium at 28 C overnight, harvested by centrifugation, washed once with wash buffer (10 mM MgCl_2_, 10 mM MES, pH 5.6), and resuspended in infiltration buffer (10 mM MgCl_2_, 10 mM MES, pH 5.6, 100 µM acetosyringone). After incubating the suspensions at room temperature for 2 hours, bacterial cultures adjusted to an OD_600_ of 0.5 were infiltrated into leaves of 4-week-old *N. benthamiana* plants using a needleless 1 mL syringe. Bioluminescence signals were captured and quantified using a Bio-Rad ChemiDoc™ Imaging System after 3 days of incubation. All primers used for plasmid construction are listed in **Supplementary Table 13**.

### Statistical analysis

All statistical analyses were performed in R (version 4.5.2). For tissue-specific expression (qPCR, n=3 biological replicates), abiotic stress time-course responses (cold, NaCl, and drought; n=3), and luciferase complementation imaging (LCI) assays (n=6), one-way analysis of variance (ANOVA) was conducted to assess overall differences among groups. When the ANOVA F-test was significant (P < 0.05), *post-hoc* pairwise comparisons were carried out using Tukey’s honestly significant difference (HSD) test. Homogeneous subsets of means were labeled with lowercase letters, where shared letters indicate no significant difference at α = 0.05. For comparison of histone H3 acetylation levels under abiotic stress conditions, relative changes were analyzed using the two-tailed Student’s t-test(n=4). Data are presented as mean ± standard deviation (SD). The R packages tidyverse, rstatix, and multcompView were used for data processing, statistical testing, and letter-based visualization.

## Results

### Genome-wide identification of *GTLC* genes in soybean

**Figure 1:**
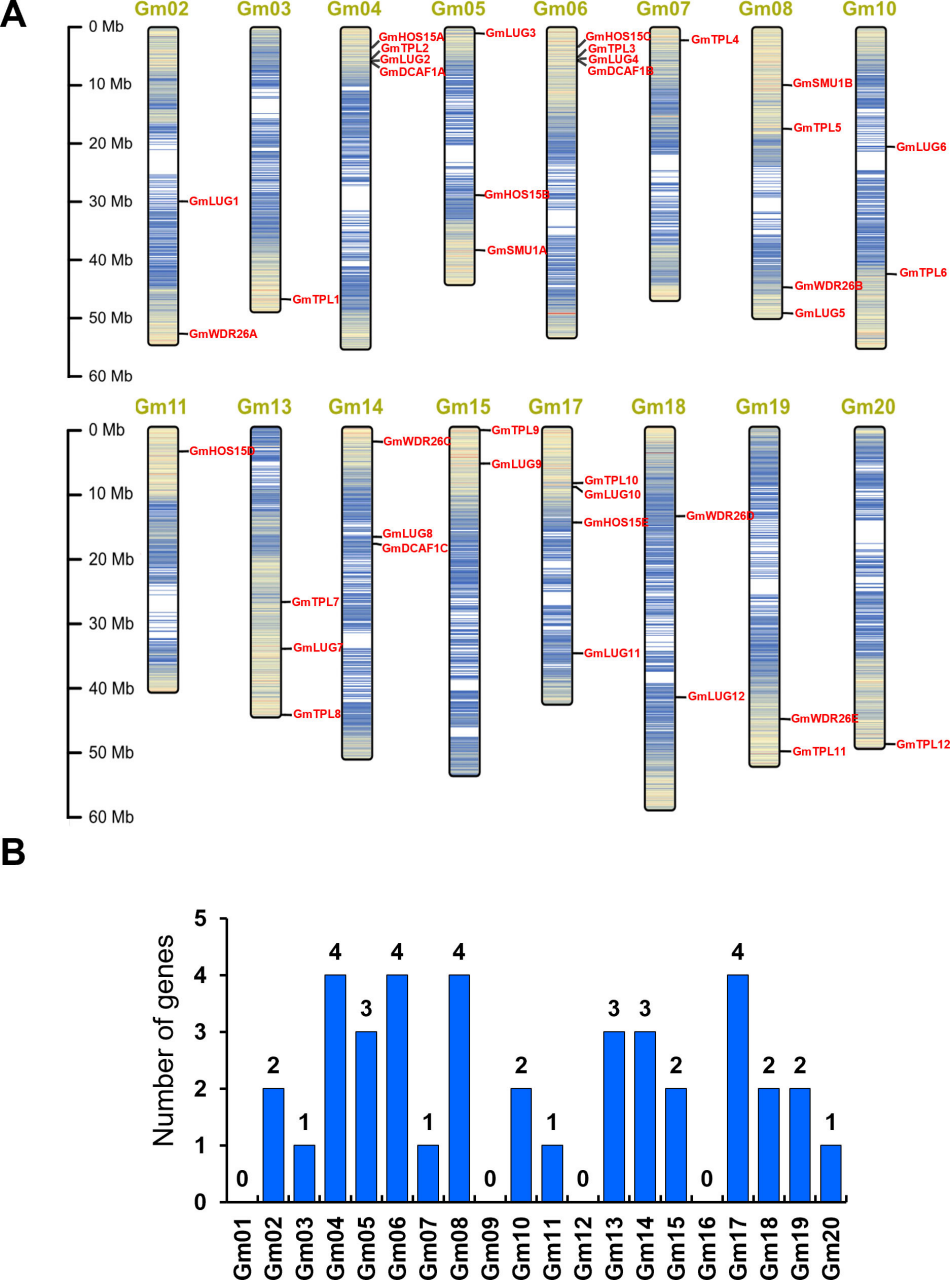
The chromosomal locations of the *GmGTLC* genes in soybean. **(A)** Distribution of all *GmGTLC* genes. **(B)** Number of *GmGTLC* genes on each chromosome.

To identify *GTLC* genes in soybean, we performed Hidden Markov Model (HMM) and BLASTp analysis using the amino acid sequences of the 13 Arabidopsis GTLC proteins, followed by selection of soybean genes encoding proteins containing LisH and WD40 domains. A total of 39 candidate *GTLC* family members were identified and named as *GmTPL1* to *GmTPL12*, *GmLUG1* to *GmLUG12*, *GmHOS15A* to *GmHOS15E*, *GmWDR26A* to *GmWDR26E*, *GmSMU1A/B*, and *GmDCAF1A* to *GmDCAF1C* based on their chromosome localization and homologous Arabidopsis genes (**Figure 1**; **Supplementary Table 1**). Chromosomal localization analysis revealed that the *GmGTLC* genes were distributed across 16 soybean chromosomes, excluding chromosomes 1, 9, 12, and 16. Four *GmGTLC* genes were located on chromosomes 4, 6, 8, and 17; three genes on chromosomes 5, 13, and 14; two genes on chromosomes 2, 10, 15, 18, and 19; and one gene on chromosomes 3, 7, 11, and 20 (**Figure 1**).

To characterize the physicochemical properties of GmGTLCs, we analyzed their amino acid sequence length, molecular weight (MW), isoelectric point (pI), instability index (II), aliphatic index (AI), grand average of hydropathicity (GRAVY), and subcellular localization (**Supplementary Table 1**). The amino acid lengths and molecular weight ranged from 513 to 1,987 residues and 57.96 to 217.41 kDa, respectively, identifying GmDCAF1A as the largest and GmSMU1A/B as the smallest protein. The pI values varied between 5.15 and 6.97, indicating that GmGTLC family proteins are generally acidic. The II ranged from 30.64 to 58.06, with GmLUG2 being the most unstable and GmHOS15A the most stable. The AI values spanned from 66.09 to 92.36, where GmWDR26A exhibited the highest and GmLUG2 the lowest values. Furthermore, the GRAVY ranged from −0.836 to −0.198, with GmHOS15B being the smallest and GmWDR26E being the largest, indicating that the GmGTLCs have high hydrophilicity. Subcellular localization predictions indicated that 27 GmGLCT members reside in the nucleus, 9 in the chloroplast, and 2 in the plastid.

### Phylogenetic tree of the GTLC family in soybean

**Figure 2:**
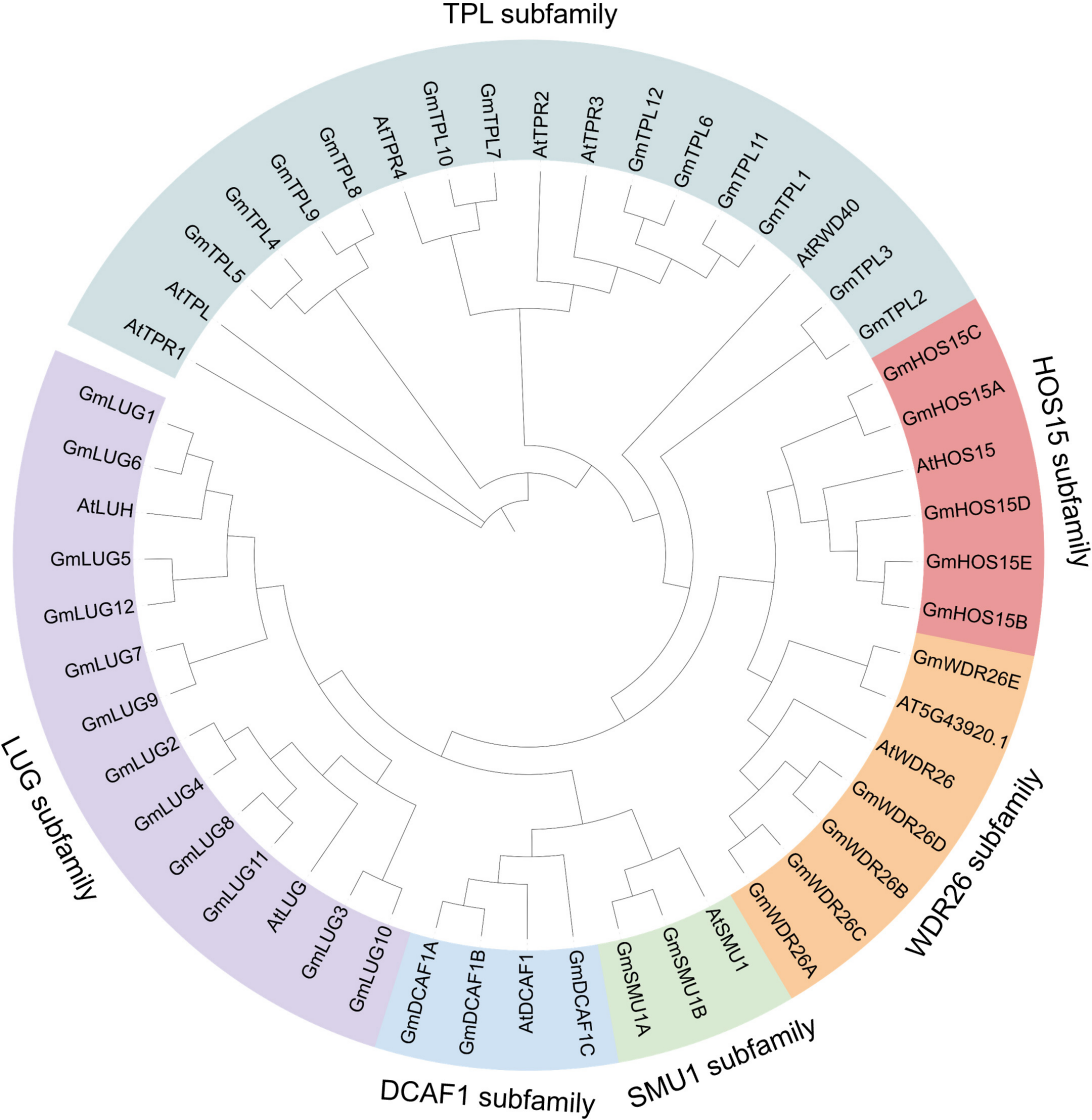
Phylogenetic analysis of GTLC family proteins from Arabidopsis thaliana and Glycine max. The six different colored backgrounds represent proteins from the six distinct GTLC subfamilies.

The proteins within the same group may share conserved structural and functional features. To elucidate the evolutionary relationships among GTLC family members, a phylogenetic tree was constructed using the full-length amino acid sequences of 13 Arabidopsis and 39 soybean GTLC proteins with IQ-Tree software (**Figure 2**). For systematic classification and the convenience of future research, the GTLC family members were categorized into six distinct subfamilies. Among these, the TPL subfamily was the largest, containing 18 members, followed by the LUG subfamily (14 members), the WDR26 subfamily (7 members), the HOS15 subfamily (6 members), the DCAF1 subfamily (4 members), and the SMU1 subfamily (3 members). Notably, each subfamily included representatives from both soybean and Arabidopsis. Given the well-documented functional roles of many Arabidopsis GTLC proteins, this phylogenetic classification provides a valuable framework for predicting the potential biological functions of GmGTLCs based on their evolutionary relationships with their Arabidopsis homologs.

### Gene structure, conserved motifs, and domains of GmGTLCs

**Figure 3:**
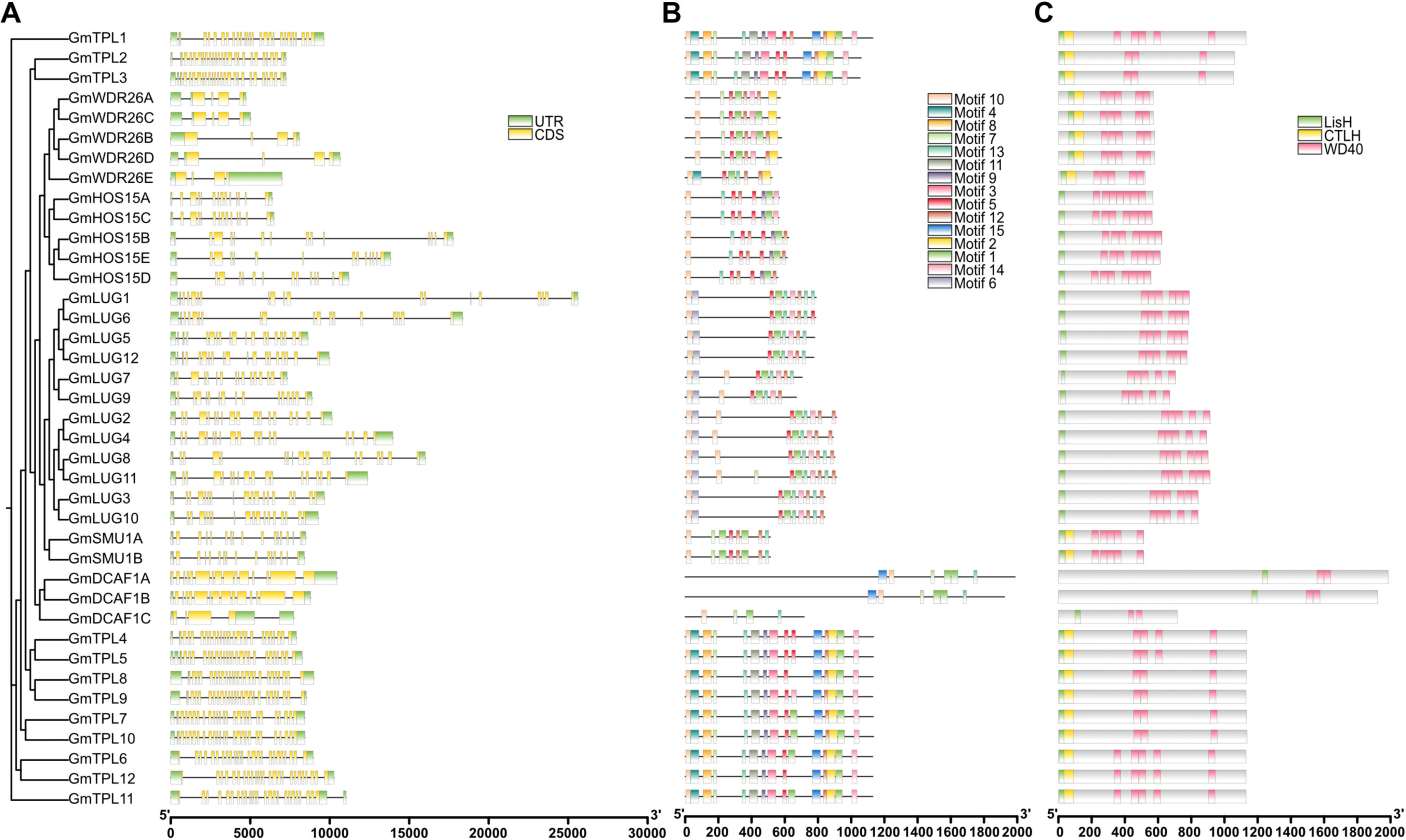
Analysis of gene and protein structure of GmGTLCs. **(A)** Gene structure of *GmGTLCs*. **(B)** Conserved motifs distribution in GmGTLC proteins. Motifs 1–15 are represented by differently colored rectangles. **(C)** Conserved domains of GmGTLC proteins. The scale bar at the bottom indicates gene/protein length.

To gain further insights into the structural features of *GmGTLC* genes and their encoded proteins, we analyzed their gene structures, conserved motifs, and protein domains. The results revealed that members within the same subfamily share highly similar patterns in terms of gene structure, motif composition, and domain organization (**Figure 3**). Gene structure analysis showed that the number of exons ranged from 4 to 25. Notably, genes belonging to the same subfamily exhibited strongly conserved structural architectures. For instance, the *TPL* subfamily generally contained 24–25 exons, the *LUG* subfamily had 16–18 exons, the *SMU1* subfamily consistently contained 16 exons, the *HOS15* subfamily comprised 14 exons, and the *WDR26* subfamily invariably contained 4 exons (**Figure 3A**; **Supplementary Table 1**). This pronounced structural conservation within subfamilies suggests a high degree of evolutionary constraint among these genes. In the motif analysis, 15 conserved motifs with lengths ranging from 21 to 50 amino acids were identified in the GmGTLC family using the online tool MEME (**Figure 3B**; **Supplementary Figure 1**; **Supplementary Table 2**). The number of motifs per protein varied from 4 to 14. Among these, three motifs, including motif 1, motif 10, and motif 13, were highly conserved across the entire GmGTLC family members. Furthermore, proteins within the same subfamily displayed relatively consistent motif compositions. For example, the TPL subfamily contained the highest number of conserved motifs (14 motifs), while the LUG, HOS15, WDR26, and SMU1 subfamilies each contained 8 conserved motifs. Domain analysis revealed that all GmGTLC proteins contain both an N-terminal LisH domain and a C-terminal WD40 domain, while homologs of TPL, WDR26, and SMU1 also possess a CTLH domain located C-terminal to the LisH domain (**Figure 3C**; **Supplementary Table 3**). Further analysis of WD40 repeat numbers indicated distinct distributions across subfamilies. The HOS15 subfamily contains 7 WD40 repeats, the LUG subfamily possesses 5 to 6 repeats, the SMU1 and WDR26 homologs contain 5 repeats, the TPL subfamily contains 3 to 5 repeats, and the DCAF1 subfamily generally comprises 2 repeats (**Figure 3C**; **Supplementary Table 3**). These findings suggest that there are potential functional similarities among the members of each subfamily.

### Cis-regulatory element analysis of promoters in *GmGTLCs*

**Figure 4:**
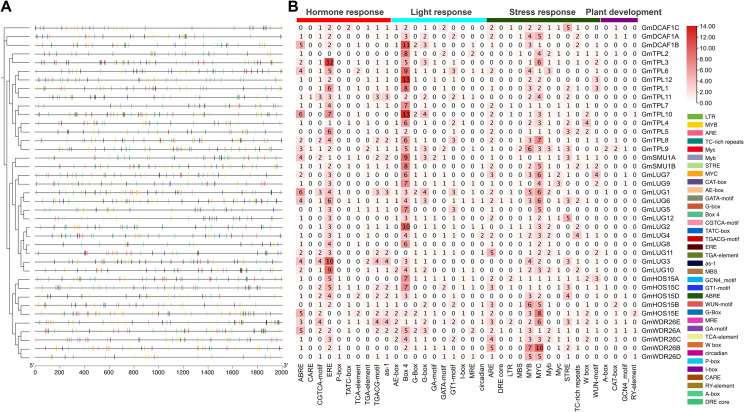
Analysis of putative cis-regulatory elements in the promoters of *GmGTLC* genes. **(A)** Locations of cis-regulatory elements in the promoter. Elements predicted within the 2000bp upstream region of the *GmGTLC* genes. Different functional categories of elements are indicated by distinct colors. **(B)** The number of cis-regulatory elements of individual *GmGTLC* genes. Numbers within boxes indicate element counts, with darker shading representing higher abundance.

Cis-regulatory elements (CRE) are specific DNA sequence motifs bound by sequence-specific transcription factors. They function as molecular switches that precisely modulate gene expression levels and spatiotemporal patterns, which is crucial for appropriate development and adaptation to environmental cues (Marand et al., 2023). To predict the transcriptional regulatory activities of the *GmGTLC* genes, the 2-kb promoter regions upstream of their start codons were analyzed using the PlantCARE database. A total of 36 functional categories of CRE were identified, including responses to hormones, light, stress, and development (**Figure 4**; **Supplementary Table 4**). Notably, the promoter regions of *GmGTLC* genes are particularly enriched in light-responsive elements, with Box4 being the most prominent. In addition, key elements involved in phytohormone responses (e.g., ERE, ABRE) and stress responses (e.g., MYB, MYC) are also abundantly present (**Figure 4B**). Strikingly, the MYC transcription factor binding site, a key element involved in stress responses, was found to be universally present in all *GmGTLC* promoters (**Figure 4B**). These results suggest that the *GmGTLC* genes may function as regulators involved in growth processes, phytohormone signaling, and stress adaptation, thereby modulating soybean development and environmental responses.

### Collinearity analysis of *GmGTLC* genes

**Figure 5:**
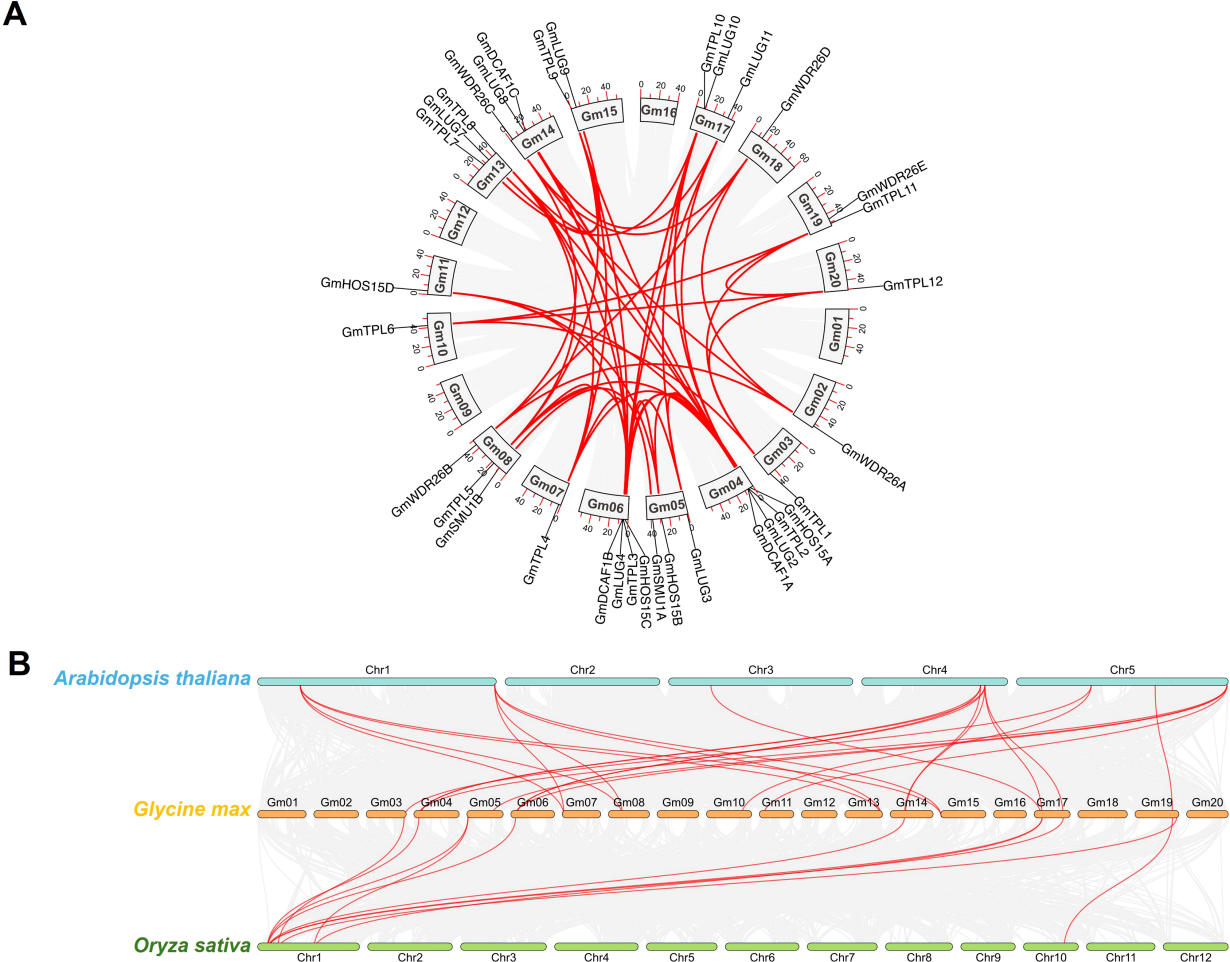
Analysis of duplication events among *GTLC* genes in soybean, Arabidopsis, and rice. **(A)** Intraspecific collinearity analysis of *GmGTLC* genes. **(B)** Interspecific comparative synteny analysis of *GTLC* genes. Red lines indicate duplicated *GTLC* gene pairs.

Collinearity analysis reveals conserved gene order and positions across species or genomic regions, thereby deepening our understanding of genome evolution and aiding in the functional prediction of genes (Wang et al., 2012, 2024). To investigate the role of gene duplication in the expansion of the *GmGTLC* gene family, we performed intraspecific collinearity analysis to identify segmental and tandem duplication events among *GmGTLC* genes. The analysis revealed 52 collinear gene pairs classified as segmental duplication, but no amplification via tandem duplication was detected (**Figure 5A**; **Supplementary Table 5**). These results suggest that segmental duplication events, rather than tandem duplication, contributed to the expansion of the *GmGTLC* gene family in soybean. To further elucidate the evolutionary relationships of *GTLC* genes across species, an interspecies comparative synteny analysis was conducted between soybean (*Glycine max*) and the dicot *Arabidopsis thaliana* or the monocot rice (*Oryza sativa*). The results revealed a closer evolutionary relationship between soybean and Arabidopsis, with 24 syntenic *GTLC* gene pairs identified, excluding those from the *SMU1* subfamily (**Figure 5B**; **Supplementary Table 6**). In contrast, only 11 syntenic gene pairs were found between soybean and rice. Notably, in rice, most orthologous pairs belonged to the *LUG* subfamily (8 pairs), and exception of the *HOS15*, *DCAF1*, and *SMU1* subfamilies (**Figure 5B**; **Supplementary Table 7**). These results provide valuable insights into the evolutionary history and potential functional similarities of the *GTLC* gene family across soybean, Arabidopsis, and rice.

To investigate the evolutionary mechanisms acting on the *GTLC* gene family, we calculated the nonsynonymous to synonymous substitution ratios (Ka/Ks) for soybean with Arabidopsis or rice duplicated gene pairs. The results showed that all *GTLC* duplicated pairs exhibited Ka/Ks ratios significantly lower than 1 (**Supplementary Tables 6**, **S7**), indicating that strong purifying selection has likely acted on these genes throughout their evolutionary history, thereby preserving their functional importance.

### Expression analysis of *GmGTLC* genes in different tissues of soybean

**Figure 6:**
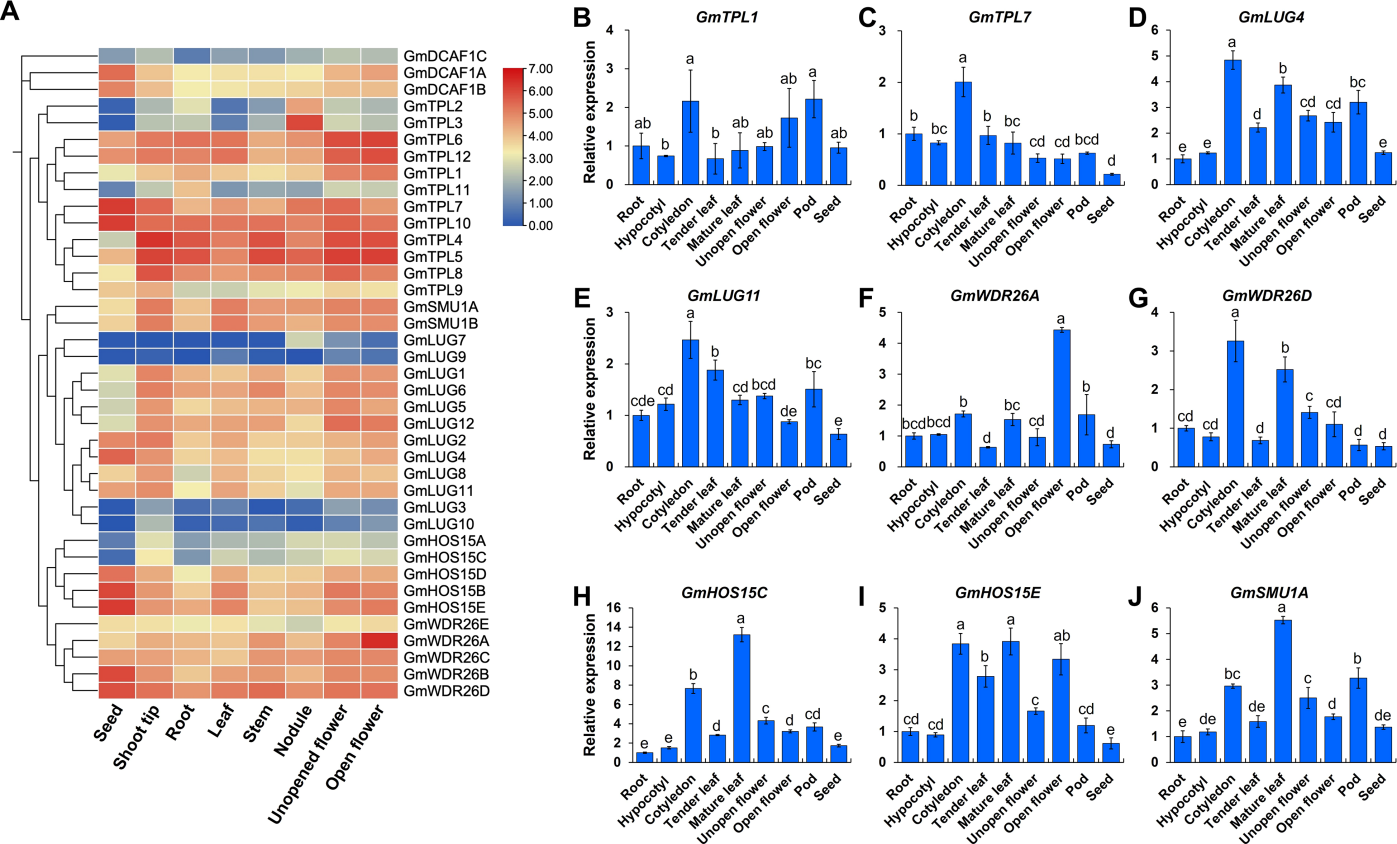
Expression patterns of *GmGTLC* genes in different tissues and organs. **(A)** Expression heatmap of *GmGTLC* genes. Expression levels are shown as log^2^-transformed TPM values, with red indicating high expression, yellow indicating moderate expression, and blue indicating low expression. **(B–J)** Relative expression levels of *GmGTLC* genes in various tissues. The expression levels of nine *GmGTLC* genes were validated by RT-qPCR, with *Cons4* used as the internal control.

To investigate the tissue-specific expression patterns of *GmGTLC* genes in soybean, we analyzed the expression levels of all identified *GmGTLCs* across multiple tissues using publicly available RNA sequencing data (Sreedasyam et al., 2023). A heatmap was constructed based on transcript levels in seeds, shoot tips, roots, leaves, stems, nodules, unopened flowers, and open flowers (**Figure 6A**; **Supplementary Table 8**). The results revealed that most *GmGTLCs* are widely expressed across all examined tissues or organs, suggesting that they may be broadly involved in soybean growth and development. In contrast, *GmLUG3,7,9,10*, Gm*HOS15A, C*, and *GmDCAF1C* showed relatively lower expression in all tissues, suggesting possible nonfunctional status or tightly regulated spatiotemporal expression. Notably, the *TPL* subfamily *GmTPL4, 5, 6, 7, 10, 12* genes displayed consistently high expression in most tissues or organs compared to other genes, implying roles in general plant growth and development. Strikingly, *GmTPL3* exhibited particularly high expression in nodules, pointing to a possible role in nodule-specific developmental programs.

To further validate the expression patterns of *GmGTLCs*, total RNA was extracted from various tissues and organs of the soybean cultivar “Williams 82”, including roots, hypocotyls, cotyledons, tender leaves, mature leaves, unopened flowers, open flowers, pods, and seeds. RT-qPCR was performed to examine the expression levels of 9 selected genes (*GmTPL1, GmTPL7, GmLUG4, GmLUG11, GmWDR26A, GmWDR26D, GmHOS15C, GmHOS15E, and GmSMU1A*) in these tissues and organs (**Figures 6B–J**; **Supplementary Table 9**). The results showed that *GmTPL1, GmTPL7, GmLUG4, GmLUG11, GmWDR26D, and GmHOS15E* were predominantly expressed in cotyledons (**Figures 6B–E, G, I**); *GmHOS15C, GmHOS15E, and GmSMU1A* were mainly expressed in mature leaves (**Figures 6H, I, J**); *GmWDR26A* and *GmHOS15E* exhibited higher expression levels in open flowers (**Figures 6F, I**). Additionally, *GmTPL1* showed increased expression not only in cotyledons but also in pods (**Figure 6B**). These findings suggest that *GmGTLC* genes are broadly expressed across soybean tissues, with some members exhibiting organ-preferential patterns, implying potential tissue-specific roles and functional redundancy.

### Expression analysis of *GmGTLC* genes under abiotic stress treatments

**Figure 7:**
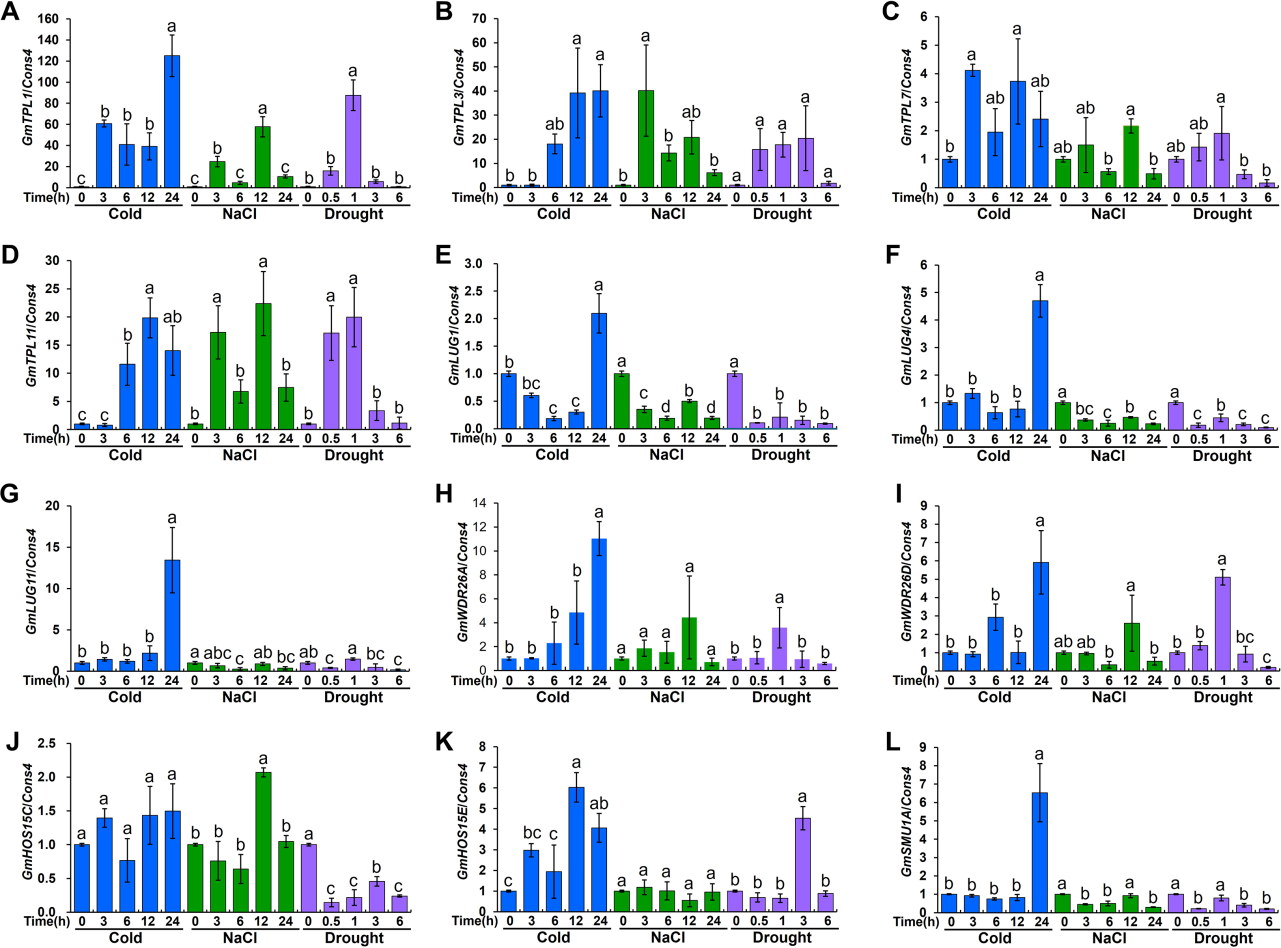
**(A–L)** Expression patterns of GmGTLC genes under cold, salt, and drought stress. Twelve selected GmGTLC genes were validated by RT-qPCR, with Cons4 used as the internal control. Different letters above bars represent significant differences at p < 0.05 (ANOVA F-test).

Previous studies have demonstrated that *GTLC* family genes play crucial roles in plant responses to abiotic stresses. To further investigate the potential functions of *GmGTLC* genes under stress conditions, 12 genes were selected considering the large number of gene families and high sequence similarity among subfamily members. Then the expression profiles were analyzed via RT-qPCR under cold (4 °C), salt (200 mM NaCl), and drought treatments (**Figure 7**; **Supplementary Table 10**; **Supplementary Figure 2**). The results showed that the expression levels of *GmGTLC* genes were significantly up-regulated or down-regulated under different stress conditions (**Figure 7**).

Under cold stress, most tested genes exhibited significant upregulation with distinct temporal expression profiles, except for *GmHOS15C* (**Figure 7J**), which was not induced by cold treatments. The expression of most genes peaked at 24 hours post-treatment, while *GmTPL7* peaked at 3 and 12 hours (**Figure 7C**), and *GmTPL11* and *GmHOS15E* peaked at 12 hours, followed by a decrease (**Figures 7D, K**). These observations suggest that the *GmGTLC* family may be broadly associated with soybean responses to cold stress. Notably, the expressions of *GmLUG1*, *GmLUG4*, *GmLUG11*, and *GmSMU1A* exhibit a sharp induction of burst specifically at 24 hours post-treatment, with no significant expression at earlier time points (3, 6, and 12 hours) (**Figures 7E–G, L**). This pattern suggests that these genes may not be linked to early cold signal responses but could be correlated with later-stage adaptation processes. Following treatment with salt, *GmTPL1*, *GmTPL3*, *GmTPL7*, *GmTPL11*, and *GmHOS15C* (**Figures 7A–D, J**) were up-regulated, with most reaching peak expression at 12 hours (*GmTPL1*, *GmTPL7*, *GmTPL11*, and *GmHOS15C*). In contrast, *GmLUG1* and *GmLUG4* were down-regulated (**Figures 7E, F**). These findings suggest that the *GmGTLC* gene family may be involved in salt stress responses, possibly through diverse expression patterns among its members. Under drought stress, the expressions of *GmTPL1*, *GmTPL7*, *GmTPL11*, *GmLUG11*, *GmWDR26A*, *GmWDR26D*, and *GmHOS15E* were up-regulated (**Figures 7A, C, D, G–I, K**). Among these, *GmTPL1*, *GmTPL7*, *GmTPL11*, *GmLUG11*, *GmWDR26A*, and *GmWDR26D* reached peak expression at 1 hour, and *GmHOS15E* peaked at 3 hours. Conversely, under the same conditions, *GmLUG1* and *GmLUG4* were down-regulated (**Figures 7E, F**), while *GmTPL3* showed no significant induction (**Figure 7B**). Taken together, these results suggest that *GmGTLC* genes may be associated with soybean responses to abiotic stresses, with diverse expression patterns that could correlate with stress adaptation.

### Abiotic stresses treatment increases histone H3 acetylation levels

**Figure 8:**
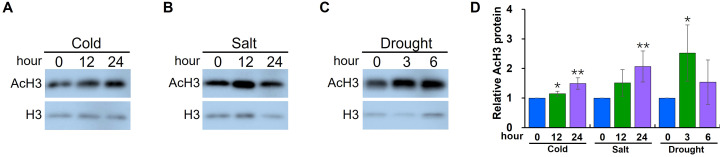
Histone H3 acetylation levels in soybean under abiotic stress conditions. **(A–C)** 15-day-old soybean plants treated with cold **(A)**, salt **(B)**, or drought **(C)** for the indicated times were analyzed by Western blot using AcH3 and H3 antibodies. **(D)** Quantification of AcH3 levels normalized to total H3, showing the fold change relative to the untreated control. Significant differences were determined by Student’s t-test (significance: *p < 0.05, **p < 0.01, n=4).

Plant stress is a challenge that disrupts development and growth, and epigenetic regulation serves as a key mechanism for stress response and adaptation (Abdulraheem et al., 2024). Transcriptional corepressors often participate in this process by recruiting HDACs to chromatin, where they repress gene expression through the modification of histone acetylation to deacetylation. To investigate the impact of abiotic stress on this epigenetic mark, we analyzed histone H3 acetylation levels in soybean plants subjected to cold, salt, and drought conditions. As shown in **Figure 8** and **Supplementary Table 11**, in 15-day-old soybean plants, the levels of histone H3 acetylation in the extracted nuclear proteins exhibit a gradual increase after 12 and 24 hours of cold treatment (**Figures 8A, D**). Exposure to salt stress also significantly increased histone H3 acetylation at 24 hours (**Figures 8B, D**). In addition, histone H3 acetylation levels were markedly elevated after 3 hours of exposure to drought stress. (**Figures 8C, D**). These findings demonstrate that abiotic stresses induce dynamic changes in histone H3 acetylation levels in soybean, highlighting the importance of this epigenetic regulatory mechanism.

### GmHOS15A and GmHOS15D interact with GmHDA12 and GmHDA13

**Figure 9:**
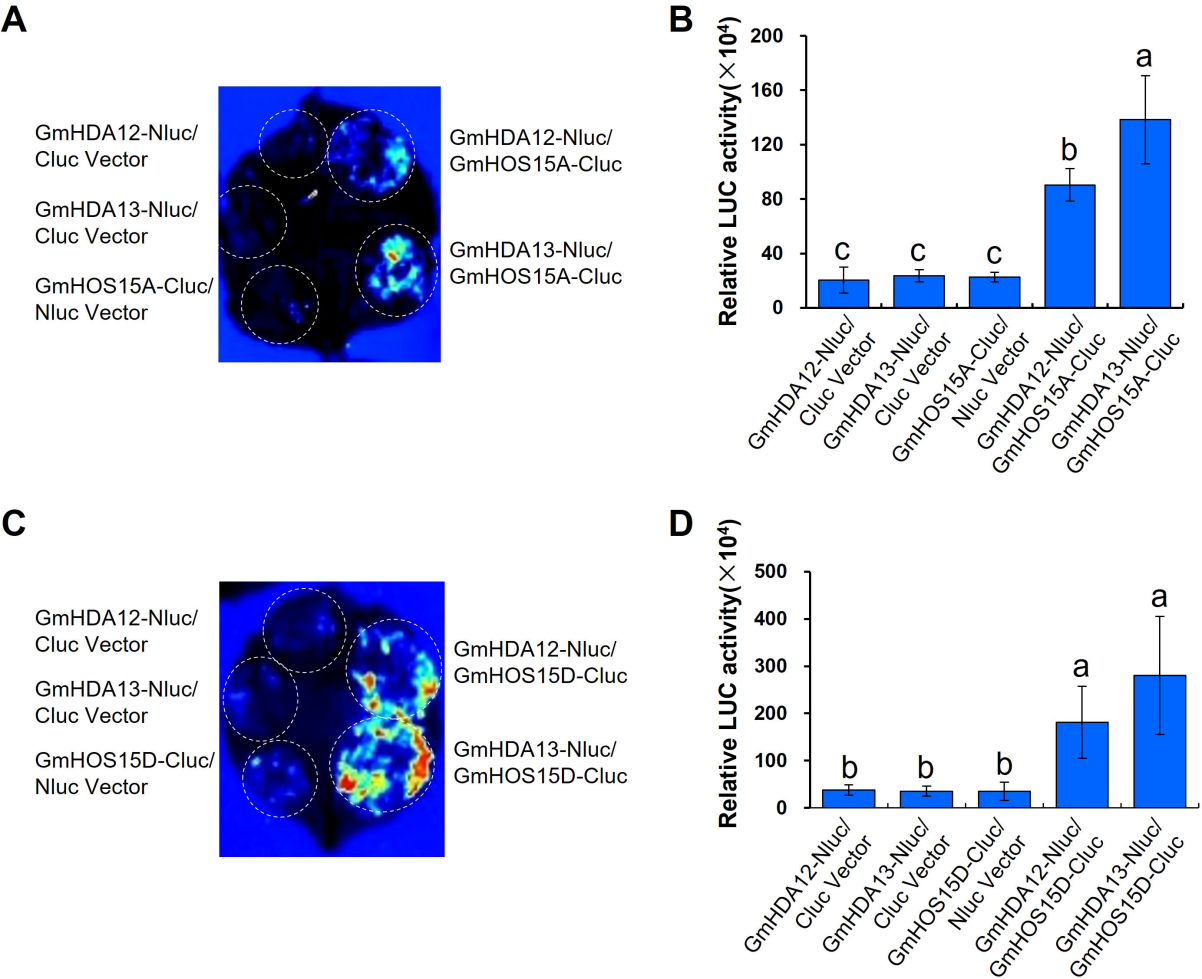
GmHOS15A and GmHOS15D interact with GmHDA12 and GmHDA13. **(A, B)** LCI assay demonstrating the association of GmHOS15A-Nluc with GmHDA12-Nluc and GmHDA13-Nluc. **(C, D)** The association of GmHOS15D-Nluc with GmHDA12-Nluc and GmHDA13-Nluc in *N. benthamiana*.

To further investigate the potential biological function of GmHOS15 proteins, we aimed to determine whether they recruit HDACs to form transcriptional corepressor complexes, thereby potentially participating in histone deacetylation. In Arabidopsis, HOS15 interacts with multiple HDACs, including HDA9, HDA6, HDA19, and HD2C (Park et al., 2018), also forms the HDA9–HOS15–PWR core HDAC corepressor complex, which plays an important role in plant development and environmental responses (de Rooij et al., 2020; Mayer et al., 2019). In soybean, there are two closely related orthologs of Arabidopsis HDA9, GmHDA12 and GmHDA13 (Yang et al., 2018). Therefore, GmHOS15A and GmHOS15D were cloned and carried out Luciferase complementation imaging (LCI) assays with these two GmHDAC proteins (**Figure 9**; **Supplementary Table 12**). Agrobacterium-mediated transient coexpression of CLuc-GmHOS15A with NLuc-GmHDA12 or NLuc-GmHDA13 in *N. benthamiana* leaves resulted in strong LUC activity compared to the negative controls of CLuc-GmHOS15A with empty NLuc vector or NLuc-GmHDA12 and NLuc-GmHDA13 with empty CLuc vector (**Figures 9A, B**). As well as, a combination of CLuc-GmHOS15D with NLuc-GmHDA12 or NLuc-GmHDA13 (**Figures 9C, D**) also showed strong LUC activity compared to the negative controls. These results suggest that, as in Arabidopsis, GmHOS15s may potentially interact with GmHDA12 and GmHDA13, possibly participating in histone deacetylation-associated regulatory processes.

## Discussion

### Identification and characterization of *GmGTLC* genes in soybean

GTLC family proteins function as key transcriptional coregulators that mediate essential functions in plant development and responses to biotic and abiotic stress through transcriptional repression. While genome-wide identification of *GTLC* gene families has been conducted in several plant species, most studies have focused exclusively on individual subfamilies such as TPL or LUG (Guan et al., 2018; Hao et al., 2014; Li et al., 2016; Yu et al., 2025; Zhang et al., 2024). To date, comprehensive analyses of the entire *GTLC* family have been reported only in Arabidopsis and maize (Li et al., 2016; Liu and Karmarkar, 2008). Recent work in soybean identified 12 *TPL*-like genes, which is consistent with our observation of a predominant *TPL* subfamily. In contrast, it reported the apparent absence of other GTLC subfamilies and did not investigate epigenetically related functions (Yu et al., 2025). This study presents the first comprehensive systematic analysis of the soybean *GmGTLC* family. A comparative framework with the model plant *Arabidopsis thaliana* was established to investigate its evolutionary conservation and diversity. Using 13 Arabidopsis GTLC protein sequences, we identified and characterized 39 *GmGTLC* family members in soybean. Phylogenetic analysis divides them into six subfamilies (TPL, LUG, WDR26, HOS15, DCAF1, and SMU1), each exhibiting high conservation in gene and protein structure among its members. These findings support the hypothesis that the *GmGTLC* gene family may have originated in ancient times and has played critical, conserved roles in plant biology. (**Figures 2**, **3**).

As a core evolutionary mechanism, gene duplication drives innovation and diversification by providing redundant gene copies that enable neofunctionalization or subfunctionalization (Saccone et al., 2025). Our collinearity analysis suggests that the core members of the *GmGTLC* gene family likely originated in a common ancestor of angiosperms. This is supported by stronger synteny between soybean and Arabidopsis, which shares 24 syntenic *GTLC* gene pairs compared to only 11 between soybean and rice (**Figure 5B**). This pattern further suggests that the orthologs may have maintained conserved roles in fundamental transcriptional repression. During soybean evolution, the family expanded not through ancient tandem or whole-genome duplication events, but primarily via repeated, lineage-specific segmental duplications, leading to the formation of the six extant subfamily members (**Figure 5A**). This mode of expansion may have enabled soybeans to finely modulate this ancient gene family, thereby driving its specific developmental programs and facilitating environmental adaptation.

Cis-acting elements function as key regulatory motifs within DNA, primarily upstream of genes, to control expression levels. Investigating these sequences is fundamental to elucidating the precise mechanisms of gene regulation (Marand et al., 2023). Our promoter analysis indicates that the regulatory regions of *GmGTLC* genes are enriched with diverse cis-regulatory elements. Notably, light-responsive elements, particularly Box4 are prominent, along with multiple elements associated with plant hormone responses (e.g., ERE, ABRE) and stress responses (e.g., MYB, MYC). The MYC transcription factor binding site, a key stress-responsive element, is present in all *GmGTLC* promoters (**Figure 4**). Together, these features may suggest a dual regulatory architecture, in which a conserved “core module” involved in general light, hormone, and stress signaling might coexist with lineage-specific elements that fine-tune responses to particular signals.

The expression profile of the soybean *GmGTLC* gene family exhibits both broad conservation and distinct tissue specificity. While most members are widely expressed across various tissues, suggesting roles in fundamental growth and potential functional redundancy (**Figure 6A**), a subset of genes demonstrates clear tissue preference. For instance, *GmTPL3* shows marked upregulation specifically in nodules, while others are enriched in cotyledons, mature leaves, or floral organs (**Figures 6B–J**). This observation suggests that the family contributes to developmental fine-tuning through spatially restricted expression patterns.

Profiling plant gene expression under stress conditions elucidates the complex regulatory networks involved in plant adaptation and stress tolerance (Wang et al., 2020). Expression analysis under stress conditions further indicates the broad involvement of *GmGTLC* genes in abiotic stress responses. Most tested genes were significantly induced under cold stress, although with distinct temporal expression profiles, some likely function in later adaptation phases rather than in the early signal transduction. Under salt and drought treatments, different family members displayed diverse expression dynamics, being either induced or repressed with varying peak times (**Figure 7**). Taken together, these findings suggest that the *GmGTLC* family coordinates soybean adaptation to environmental stresses through a complex regulatory network, the operation of which is specific to both timing and stress type. However, these conclusions are based on transcriptional profiling, but direct functional evidence is required to confirm causality.

### Understanding GTLC family function through histone acetylation

In this study, we observed that cold, salt, and drought stresses induced significant increases in global nuclear histone H3 acetylation levels in soybean (**Figure 8**). Although these findings suggest that abiotic stresses dynamically regulate this epigenetic mark, a key limitation of the present study should be acknowledged. Western blot analysis reflects global changes in histone H3 acetylation but lacks site-specific chromatin information. Therefore, our data cannot directly determine how GTLCs regulate histone deacetylation at specific genomic loci during stress responses. It is worth noting that some studies have reported that the Arabidopsis GTLCs are also involved in regulating the activation of target gene expression and may even function as a transcriptional activator under certain conditions (You et al., 2019; Shen et al., 2020; Shi et al., 2024). This suggests that the role of this family may not be confined to transcriptional repression, and its precise function in transcriptional regulation warrants further investigation. Future studies should generate mutant or overexpression lines of soybean of *GTLC* family genes and employ locus-specific methods, such as chromatin immunoprecipitation (ChIP) analysis, to investigate whether GTLC target gene promoters change in histone acetylation/deacetylation and GTLC binding status. Special attention should be paid to acetylation sites, including H3K9, H3K14, and H3K27, which are known to be regulated by the GTLC family in Arabidopsis and other plants (Shrestha et al., 2014; Deng et al., 2022; Zareen et al., 2022; Shi et al., 2024; Yusuf et al., 2025), as well as to the potential synergistic effects between different histone modification mechanisms and acetylation. Addressing these issues will help to more accurately elucidate how epigenetic regulation promotes soybean adaptation to abiotic stress.

### GmHOS15 may facilitate transcriptional repression by recruiting GmHDAC for epigenetic regulation in soybean.

In Arabidopsis, the repressor activity of LUG is dependent on HDAC activity and its direct interaction with HDA19 (Gonzalez et al., 2007). Similarly, TPL interacts with HDA19, and BES1 forms a complex that mediates the epigenetic repression of *ABI3*, thereby attenuating ABA signaling (Ryu et al., 2014). HOS15 participates in various stress responses and developmental processes, including plant immunity, photoperiodic flowering, leaf senescence, miRNA biogenesis, and adaptation to cold and drought (Ali et al., 2025). During development, and in response to cold stress, ABA signaling, and immune responses, HOS15 likely recruits and interacts with HDA9 to form a transcriptional repressor complex, thereby mediating the repression of target gene expression (Mayer et al., 2019; Park et al., 2019, 2023, 2018; Shen et al., 2020; Yang et al., 2020). In this study, based on amino acid sequence comparison, we identified five homologs of Arabidopsis HOS15 in soybean (GmHOS15A–E) and attempted to clone all five genes. However, due to their high sequence similarity, we successfully cloned only *GmHOS15A* and *GmHOS15D*. Protein–protein interaction assays in revealed that both GmHOS15A and GmHOS15D interact with GmHDA12 and GmHDA13(**Figure 9**). Although these interactions were observed in a heterologous system, they may indicate that, similar to the mechanism in Arabidopsis, GmHOS15 proteins in soybean associate with HDACs and potentially assemble into a transcriptional corepressor complex, thereby potentially contributing to developmental and stress-responsive epigenetic regulation. Nevertheless, the functional relevance of these interactions in soybean and their direct contribution to stress-induced epigenetic changes remain to be demonstrated.

### Potential corepressor activity of soybean DCAF1 homologs

In Arabidopsis, DCAF1 has been characterized as a substrate receptor for CUL4-DDB1-based E3 ubiquitin ligases, playing crucial roles in plant developmental processes and abscisic acid signaling (Zhang et al., 2008; Seo et al., 2014; Peng et al., 2022). Notably, *DCAF1* encodes a middle-positioned LisH domain, which distinguishes it from other *GTLC* subfamily members that typically contain an N-terminal LisH domain. Furthermore, its potential role as a transcription corepressor remains unclear, as this function has not been experimentally confirmed. Our study identified three DCAF1-like genes in soybean. Nevertheless, the potential transcriptional corepressor activity of these soybean homologs requires further investigation.

### Future perspectives and research directions

Although this study provides some useful theoretical insights for future research, several limitations should be noted. First, functional predictions for the GmGTLC family are primarily based on bioinformatics analysis, and their biological significance in soybean requires further experimental validation. Second, although we confirmed the interaction between the GmHOS15 protein and histone deacetylases (GmHDAC) using a heterologous expression system, we did not provide direct causal evidence in soybean, nor did we clarify whether and how GmHOS15 affects the expression of specific target genes or the status of particular histone modification sites under abiotic stress conditions. Third, functional validation has so far been limited to a few GmGTLC members, and the functions of other family members remain to be investigated. Fourth, the hypothesis that GmGTLC acts as an epigenetic corepressor in stress responses remains speculative. To overcome these limitations, functional validation is required, including direct protein–protein interaction assays, ChIP analysis, phenotypic analysis of mutant and overexpression lines, transcriptomic or ChIP-seq profiling, and direct measurement of histone acetylation levels at target loci.

Furthermore, several key questions remain to be addressed, including how GmGTLC participates in signal transduction pathways, responds to endogenous and exogenous signals, identifies its upstream regulators, epigenetically represses the transcription of downstream target genes, interacts with associated proteins, and is regulated by post-translational modifications that may influence its activity. Addressing these questions will also require the generation of GmGTLC knockout and overexpression transgenic lines for comprehensive functional characterization. In addition, field trials under diverse environmental conditions will be essential to validate the role of GmGTLC in yield stability and stress responsiveness.

## Conclusions

In this study, we present the first genome-wide identification and comprehensive characterization of the GmGTLC family in soybean. Our phylogenetic, structural, and promoter analyses revealed their evolutionary conservation and potential functional similarities within each subfamily. Expression profiling demonstrates that *GmGTLC* genes are differentially regulated under abiotic stresses, with most members responding to cold treatment. The observed increase in histone H3 acetylation under stress conditions highlights the role of epigenetic mechanisms in soybean stress adaptation. Furthermore, validation of the interactions between GmHOS15 proteins and GmHDA12 and GmHDA13 suggests the presence of a potential functional GmHOS15–GmHDAC corepressor complex that may play an important role in controlling histone H3 acetylation status. Collectively, these findings may provide a preliminary basis for understanding the potential epigenetic regulatory mechanisms mediated by GmGTLCs in soybean development and stress responses.

## Data Availability

The transcriptome expression data analyzed in this study are from a publicly accessible repository (NCBI BioProject, accession number PRJNA372533).

## Supplementary material

Supplementary Table 1 Basic physicochemical properties of GmGTLCs.

Supplementary Table 2 The motifs in GmGTLC proteins.

Supplementary Table 3 The domain distribution of GmGTLC proteins.

Supplementary Table 4 Cis-regulatory element analyses of the *GmGTLC* gene promoter regions.

Supplementary Table 5 The duplication events analysis of *GmGTLC* genes.

Supplementary Table 6 The duplication events analysis of *GTLC* genes in *Glycine max* and *Arabidopsis thaliana*.

Supplementary Table 7 The duplication events analysis of *GTLC* genes in *Glycine max* and *Oryza sativa*.

Supplementary Table 8 Tissue−specific expression profiles of *GmGTLC* genes derived from public RNA−seq data.

Supplementary Table 9 RT-qPCR analysis of nine selected *GmGTLC* genes across different soybean tissues and organs.

Supplementary Table 10 RT-qPCR analysis of twelve *GmGTLC* genes in soybean under cold, salt, and drought stress conditions.

Supplementary Table 11 Quantification data for histone H3 acetylation.

Supplementary Table 12 LCI assays of GmHOS15 and GmHDAC in *N. benthamiana*.

Supplementary Table 13 Primers used in this study.

Supplementary Figure 1 The motif logo of GmGTLC proteins.

Supplementary Figure 2 Morphological phenotypes of soybean seedlings exposed to salt and drought stress.
