# Supplementary figures and images for: Genome-wide identification and characterization of the Groucho/Tup1-like corepressor family identifies a potential role in the epigenetic regulation of abiotic stress responses in soybean
Source: Front Plant Sci. 2026 Jun 4;17:1825108. doi: 10.3389/fpls.2026.1825108 (PMC13275459; doi:10.3389/fpls.2026.1825108)

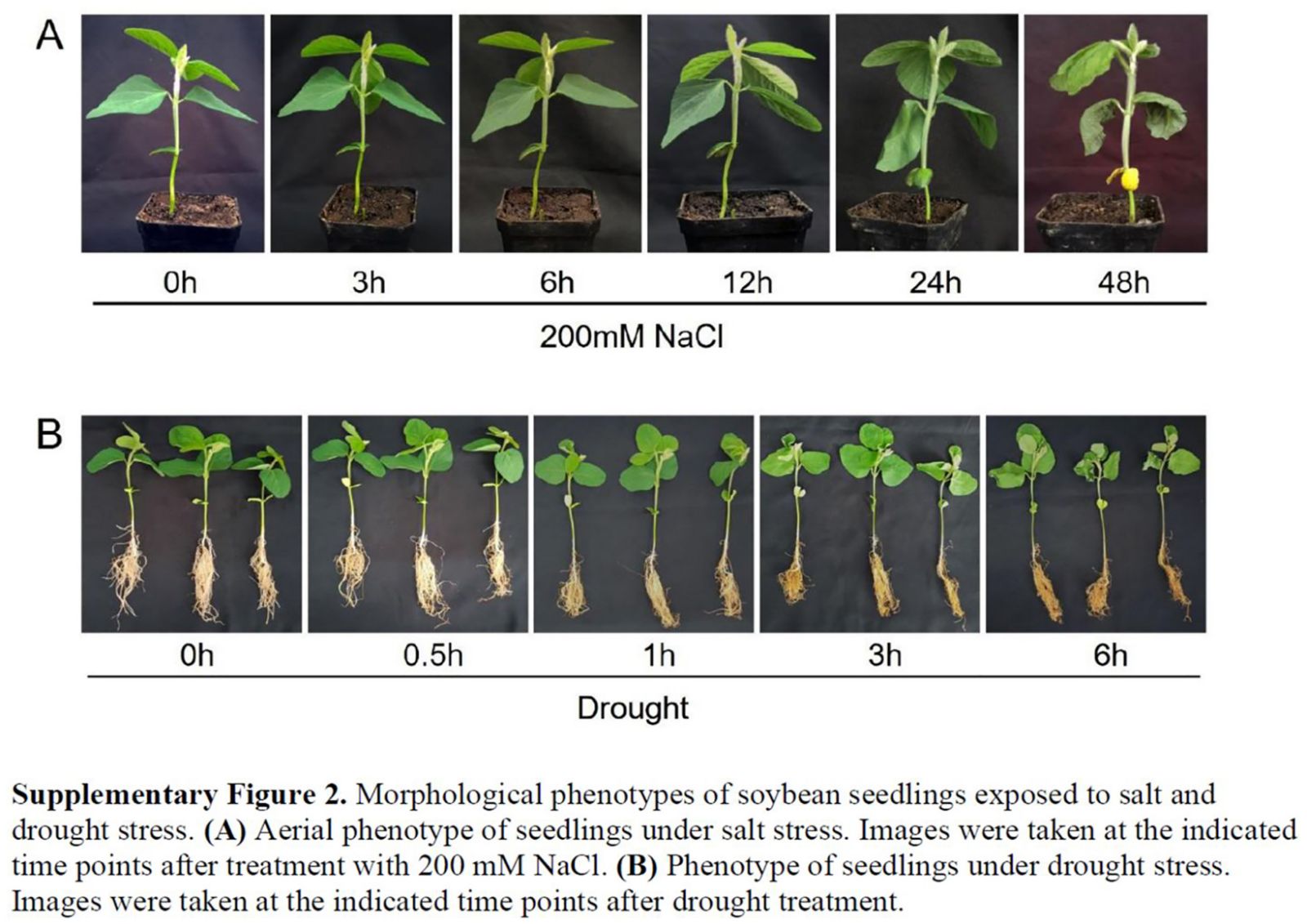

Supplement: Supplementary Figure 2 — Morphological phenotypes of soybean seedlings exposed to salt and drought stress. [file Image2.jpg]
